# The AxBioTick Study: *Borrelia* Species and Tick-Borne Encephalitis Virus in Ticks, and Clinical Responses in Tick-Bitten Individuals on the Aland Islands, Finland

**DOI:** 10.3390/microorganisms11051100

**Published:** 2023-04-22

**Authors:** Nellie Carlströmer Berthén, Eszter Tompa, Susanne Olausson, Clara Nyberg, Dag Nyman, Malin Ringbom, Linda Perander, Joel Svärd, Per-Eric Lindgren, Pia Forsberg, Peter Wilhelmsson, Johanna Sjöwall, Marika Nordberg

**Affiliations:** 1 Borrelia Research Group of the Aland Islands, 22100 Mariehamn, The Aland Islands, Finland; 2 Bimelix AB, 22100 Mariehamn, The Aland Islands, Finland; 3 Department of Biomedical and Clinical Sciences, Division of Inflammation and Infection, Linkoping University, 581 83 Linkoping, Sweden; 4 The Aland Islands Healthcare Services, 22100 Mariehamn, The Aland Islands, Finland; 5 Clinical Microbiology, Laboratory Medicine, County Hospital Ryhov, 551 85 Jonkoping, Sweden; 6 Department of Infectious Diseases, Vrinnevi Hospital, 603 79 Norrkoping, Sweden

**Keywords:** *Borrelia*, tick-borne encephalitis virus, *Ixodes ricinus*, tick bite, clinical, prospective study, ELISA, antibody responses, clinical responses

## Abstract

The AxBioTick study was initiated to investigate the prevalence of ticks and tick-borne pathogens and their impact on antibody and clinical responses in tick-bitten individuals on the Aland Islands. This geographical area is hyperendemic for both Lyme borreliosis (LB) and Tick-borne encephalitis (TBE). Blood samples and ticks were collected from 100 tick-bitten volunteers. A total of 425 ticks was collected, all determined to *Ixodes ricinus* using molecular tools. Of them 20% contained *Borrelia* species, of which *B. garinii* and *B. afzelii* were most common. None contained the TBE virus (TBEV). Blood samples were drawn in conjunction with the tick bite, and eight weeks later. Sera were analyzed for *Borrelia*- and TBEV-specific antibodies using an ELISA and a semiquantitative antibody assay. In total 14% seroconverted in *Borrelia* C6IgG1, 3% in TBEV IgG, and 2% in TBEV IgM. Five participants developed clinical manifestations of LB. The high seroprevalence of both *Borrelia* (57%) and TBEV (52%) antibodies are likely attributed to the endemic status of the corresponding infections as well as the TBE vaccination program. Despite the similar prevalence of *Borrelia* spp. detected in ticks in other parts of Europe, the infection rate in this population is high. The AxBioTick study is continuing to investigate more participants and ticks for co-infections, and to characterize the dermal immune response following a tick bite.

## 1. Introduction

Tick-borne diseases (TBDs) are an increasing concern for public health, especially in highly endemic areas such as the Aland Islands. This archipelago is part of Finland, located in the Baltic Sea between the mainland of Finland and Sweden. It has a high incidence of Lyme borreliosis (LB) and Tick-borne encephalitis (TBE) [1,2]. In 2021, the incidence of LB on the Aland Islands was estimated to be 1713/100,000 inhabitants, which is approximately 36 times higher than in mainland Finland. Similarly, the incidence of TBE was around six times higher than in mainland Finland with a 5-year estimation of 41 cases/100,000 inhabitants/year (2017–2021) [3,4].

Previous studies have shown that the Aland population has a high seroprevalence of both *Borrelia* C6 IgG/IgM antibodies and *Borrelia* IgG antibodies compared to other European populations. A study where blood samples from tick-bitten individuals were collected from 2008–2009 showed that 43% of the Aland population had detectable *Borrelia* C6 IgG/IgM antibodies, which is higher than in Sweden; 35%. Furthermore, *Borrelia* IgG antibodies were detected in 23% of the Aland population, compared to 17% in Sweden and 4.2–13% in some other European countries [1,5,6,7,8,9,10]. The seroprevalence of TBE-virus (TBEV) IgG antibodies is 0.4–5.3% in nearby geographical areas such as Norway and Sweden where TBE vaccination is still uncommon for the general population [11,12]. This was also the case for the Aland population where the seroprevalence was 6.0% in 1995, before the vaccination program. Since 2006, three doses of the TBE vaccine offered free of charge to all inhabitants of the Aland Islands, has led to an increase in the seroprevalence to 88% in 2018 among adults [13]. Despite the vaccination program, the incidence of TBE is still substantial.

The *Borrelia (B.)* species belonging to the *B. burgdorferi* sensu lato (B.b.sl) complex are the most common tick-borne human pathogens in the Northern Hemisphere. Several species of B.b.sl. cause LB, a multi-systemic inflammatory disease that can affect different organs with multiple clinical manifestations [14]. At least four European species in the B.b.sl complex are considered human-pathogenic, i.e., *B. burgdorferi sensu stricto*, *B. afzelii, B. garinii,* and *B. bavariensis* [15]. Species that can potentially cause LB include *B. spielmanii*, *B. bisettii*, *B. lusitaniae,* and *B. valaisiana* [16]. It has been reported that around 22% of detached ticks from the Aland Islands contain B.b.sl. [5]. A somewhat higher prevalence has been observed in ticks collected in Sweden (27%) and in Finland (32%) [5,17].

TBEV is a flavivirus, able to infect the central nervous system. Around 90–95% are asymptomatic but one-third of all infected patients develop severe neurologic symptoms. Patients who have neurological involvement often suffer from long-term complications [18]. There are three different subtypes of TBEV of which the European subtype was discovered on the island of Kumlinge in the Aland archipelago in 1959 and later isolated in 1962 [19]. The mortality of infections caused by the European subtype of TBEV is around 1–2% [18]. The prevalence of TBEV in ticks detached from humans is low on the Aland Islands (0.4%), in Sweden (0.2%), and in ticks collected with the cloth-dragging method in Finland (0%) [2,17].

Altogether, the high incidence of LB and TBE, the TBEV vaccination program, and long persisting antibodies have resulted in high seroprevalences in the Aland population, which causes diagnostic difficulties in determining whether seropositivity depends on previous exposure, ongoing infection, or vaccination responses. Despite extensive scientific efforts to understand the specific disease mechanisms involved, the contributing factors of developing disease after a tick bite, the clinical response of TBDs, and the progression to severe disease remain largely unknown. Since the initiation of the TBD STING-study in 2007 [20], no studies on the prevalence of TBDs and clinical manifestations of the bitten participants have been conducted on the Aland Islands. This collaborative effort between Sweden and the Aland Islands contributed to many important insights into TBDs. The lessons learned from the TBD STING-study gave rise to many further questions about the local immunological responses after tick bites which the AxBioTick study aims to investigate. The overall purpose of the study is to identify contributing factors important for the development of TBDs on the Aland Islands and to further understand how the human immune system responds to tick bites and tick-borne pathogens (TBPs). 

The specific aims of the present paper are to (1) present the design of the AxBioTick study, (2) to determine the prevalence of *Borrelia* spp. and TBEV in ticks that have bitten humans, (3) to characterize the detached ticks regarding species, developmental stage, and blood-feeding time, (4) to relate the tick characteristics to the clinical response of the participants and, (5) to correlate the antibody response in participants to clinical manifestations of LB and TBE.

## 2. Materials and Methods

### 2.1. Study Design

**Figure 1:**
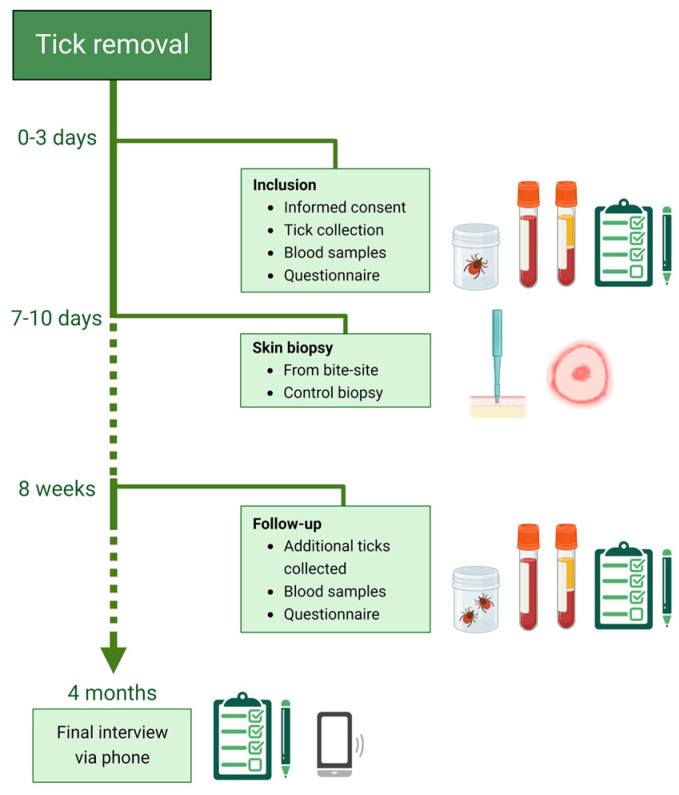
Flow chart of the AxBioTick study design. Inclusion occurs within three days after the tick bite when the tick is collected, blood samples are drawn, and a questionnaire is filled in. Seven to ten days later a skin biopsy is taken from the site of the tick bite. After eight weeks (+/− 2 weeks), additionally attached ticks are collected, blood samples are drawn and a questionnaire regarding new symptoms is filled in. A final follow-up after four months takes place by phone.

The AxBioTick study is a prospective, ongoing observational study, initiated in August 2018. Tick-bitten individuals are recruited through advertisements in local newspapers, on local radio, and on posters in public spaces. At the first visit, the tick and blood samples are collected (Figure 1). A questionnaire is filled in regarding the participant’s general condition, previous TBDs, and location of the tick bite on the body and the time point the participant noticed the tick. Informed consent is signed by the participant. Skin punch biopsies are taken 7–10 days after inclusion. After a further eight weeks (+/− 2 weeks), additional blood samples are taken, and additional detached ticks are collected. A second questionnaire regarding health status and symptoms is filled in. A concluding follow-up via phone is performed after four months, including questions about additional tick bites and symptoms. Participants who develop clinical symptoms during the study period are further investigated and diagnosed by a physician according to clinical routines.

Inclusion criteria are age >18 years and living on the Aland Islands at the time of the recruitment. The exclusion criteria are ongoing treatment with immunosuppressive or immunomodulatory drugs, cytostatic agents, radiation therapy, anti-coagulants, antibiotics, and current tick-borne or other infections.

### 2.2. Handling and Storage of Ticks and Blood Samples

On the day of collection, the ticks are frozen at −75 °C. Serum is collected using silicon dioxide (SiO_2_) tubes to initiate coagulation. The serum samples are centrifuged approximately two hours after collection and then stored at −75 °C within five hours after collection. 

### 2.3. Tick Collection and Developmental Stage

The ticks were photographed dorsally and ventrally using a USB microscope (Dino-Lite 90X, Dino-Lite Europe, Almere, The Netherlands), and the life stage and sex of adult ticks were morphologically determined before extraction of total nucleic acid (NA). The feeding time was estimated using the software DinoCapture 2.0 (Dino-Lite Europe) based on the photographs. Obtained measurements were used to calculate the scutal index (SI) and coxal index (CI) for each tick as described by Gray et al. [21]. 

### 2.4. Extraction of Nucleic Acid from Ticks and Synthesis of cDNA

The nucleic acid (NA) was extracted together with two positive controls. *B. burgdorferi* sensu stricto strain B31 (ATCC 35210, 108 cells/mL) and inactivated TBEV antigen strain K23 (Encepur^®^, Chiron Vaccines, Marburg, Germany) were used as positive controls. RNase-free water was used as a negative control. Following this, one of two methods was used. The first 59 samples were extracted using AllPrep DNA/RNA Mini Kit (Qiagen, Singapore). In this method, ticks were individually placed in a 2 mL tube with 350 µL RLT buffer, 1% ꞵ-mercaptoethanol, and a 5 mm steel bead. The mixture was homogenized using a BeadBug™ 6 microtube homogenizer (Benchmark Scientific, Sayreville, NJ, USA) for 2 min at 25 Hz. The tube was then centrifuged for 3 min at 20,000× *g* and the supernatant was transferred to a new tube. Finally, the RNA and DNA were extracted according to the manufacturer’s instructions. 

The remaining 366 samples were extracted using alphaClean Mag RNA/DNA Kit (Microgen, Neuried, Germany). The ticks were individually placed in a 2 mL tube with 500 µL Binding buffer (P1), 50 µL Proteinase [20 mg/mL], 4 µL PolyA/Carrier RNA (PA), and a 5 mm steel bead and homogenized using a BeadBug™ 6 microtube homogenizer (Benchmark Scientific, Sayreville, NJ, USA) for 2 × 1.5 min on 25 Hz. The tube was then centrifuged for 3 min at 13,250× *g*. and 500 µL of the supernatant was transferred to a new tube for total NA extraction according to the manufacturer’s instructions. The two extraction methods described above were tested to be equally comparable.

According to the manufacturer’s instructions, the eluted NA from both methods was reverse-transcribed to cDNA using an Illustra™ Ready-to-Go RT-PCR Beads kit (GE Healthcare, Amersham Place, UK) according to manufacturer’s instructions. The final product of 50 µL cDNA was frozen at −80 °C.

### 2.5. Molecular Tick Species Determination

Tick species were determined using a duplex TaqMan qPCR assay with primers specific for *Ixodes (I.) ricinus* and *I. persulcatus* as previously described by Sormunen et al. [22]. The primers IXO-I2-F4 and IXOI2-R4 are designed to target the internal transcribed spacer (ITS2) gene in *Ixodes* spp. (Table 1). The probes Ipe-I2-P4 and Iri-I2-P4 are designed to target *I. persulcatus* and *I. ricinus*, respectively (Table 1).

Each 20 µL reaction consisted of 10 µL Maxima^®^ Probe qPCR Master Mix (2X) (Thermo Fisher Scientific, Waltham, MA, USA), 0.4 µL of each primer (10 µM; Invitrogen, Waltham, MA, USA), 0.3 µL of probe Iri-I2-P4 (10 µM, Invitrogen), 0.2 µL of probe Ipe-I2-P4 10 µM, (Invitrogen), 6.7 µL RNase-free water and 2 µL cDNA template. A C1000™ Thermal Cycler, CFX96™ qPCR detection system (Bio-Rad Laboratories, Inc., Hercules, CA, USA) was used to perform the assay. An activation step at 95 °C for 5 min was followed by 45 cycles of 95 °C for 10 s and 60 °C for 1 min. RNase-free water was used as a negative control. 

The samples where the species could not be determined using the qPCR assay were further analyzed. In these samples, the 16S rDNA gene was amplified according to a protocol described by Halos et al. [23].

### 2.6. Molecular Detection of Borrelia spp and Determination of Species

An in-house, genus-specific qPCR assay targeting the *Borrelia* spp. 16S rRNA was used to detect the pathogen (Table 2) as previously described by Gyllemark et al. [24]. Each 20 µL reaction contained 10 µL Maxima^®^ Probe qPCR Master Mix (2X) (Thermo Fisher Scientific), 0.4 µL of each primer and probe (10 µM, Invitrogen), 2 µL template cDNA, and 6.8 µL RNase-free water. The PCR reactions were performed on a LightCycler 480 (Roche Applied Science, Penzberg, Germany). The protocol used was as follows: 95 °C for 5 min, 50 cycles of 95 °C for 10 s, and 60 °C for 1 min. As a positive PCR control, cDNA samples positive for *B. afzelii* strain ACA-1 confirmed by sequencing in an earlier study were used. RNase-free water was used as a negative control. 

Positive samples were further analyzed with a *B. miyamotoi*-specific TaqMan real-time PCR assay as described by Hovius et al. [25]. The primers Bm_F and Bm_R, and the probe Bm_P are designed to target the *B. miyamotoi* flagellin B gene (*flaB*) (Table 2). Each 20 µL reaction consisted of 10 µL Maxima^®^ Probe qPCR Master Mix (2X) (Thermo Fisher Scientific), 0.4 µL of each primer (10 µM, Invitrogen), and probe (10 µM, Life Technologies, Delhi, India), 3.8 µL RNase free water, and 5 µL cDNA template. The PCR reactions were performed on an A C1000™ Thermal Cycler, CFX96™ RealTime PCR Detection System (Bio-Rad Laboratories Inc.); 95 °C for 10 min, 45 cycles of 95 °C for 5 s, 60 °C for 35 s, and a final cycle of 37 °C for 20 s. A synthetic plasmid containing the target sequence for the assay was used as a positive control. The plasmid was synthesized and cloned into Eurofins standard vector carrying the ampicillin selection marker (Eurofins Genomics, Ebersberg, Germany). RNase-free water was used as a negative control. 

To determine the species of *B. b.*sl. in the samples positive in the real-time PCR assay, a nested conventional PCR assay previously described by Postic et al. [26] was used. The primers B5S-23S_F and B5S-23S_R targeting the intergenic variable spacer region (IGS) between the 3’ end of the 5S rRNA and the 5’ end of the 23S rRNA (Table 2), were used. For one 50 µL reaction 5 µL of 10 Phusion™ HF Buffer (Thermo Fisher Scientific), 1 µL of dNTP, 1 µL of each primer (10 µM, Invitrogen), 0.38 µL of Phusion™ HF DNA polymerase (Thermo Fisher Scientific), 5 µL of the template, and 36.62 µL RNase-free water were used. The assay was performed on a Bio-Rad MyCycler™ thermal cycler (Bio-Rad Laboratories Inc.) using the following protocol: 95 °C for 5 min, 39 cycles of 95 °C for 15 s, 57 °C for 30 s, and 72 °C for 30 s, 72 °C for 7 min. The second PCR mixture contained 5 µL of the obtained PCR product from the first assay. The primers B5S-23S_Fn and B5S-23S_Rn (10 µM, Invitrogen, Table 2), previously described by Wilhelmsson et al. [27], were used, and the number of cycles was increased to 42.

The PCR products were sent to Macrogen Europe BV (Amsterdam, The Netherlands) for Sanger Sequencing. The sequences were confirmed by sequencing both strands. The obtained chromatograms were initially edited and analyzed using BioEdit Software v7.0 (Tom Hall, Ibis Therapeutics, Carlsbad, CA, USA) and then compared to existing sequences in the Basic Local Alignment Search Tool (BLAST).

### 2.7. Molecular Detection of TBEV

A duplex TaqMan PCR assay was used to detect TBEV as previously described by Schwaiger and Cassinotti, and Gäumann et al. [28,29]. All the primers and probes target regions in the Neudoerfl strain (U27495) of TBEV that are present in all three subtypes of TBEV. For one 20 µL reaction, 10 µL Maxima^®^ Probe qPCR Master Mix (2X) (Thermo Fisher Scientific), 0.4 µL of each primer and probe (10 µM, Invitrogen), 5.6 µL RNase-free water, and 2 µL cDNA template were used. A C1000™ Thermal Cycler, CFX96™ Real-Time PCR Detection System (Bio-Rad Laboratories, Inc.) was used to perform the assay with the following protocol: 95 °C for 5 min activation step, followed by 45 cycles of 95 °C for 10 s and 60 °C for 1 min. 

### 2.8. Detection of Borrelia-C6IgG1 Antibodies

Serum samples drawn at inclusion (sample 1) and eight weeks (sample 2) after inclusion were analyzed in parallel with an in-house enzyme-linked immunosorbent assay (ELISA) to detect IgG1 antibodies against the *Borrelia* specific C6 peptide (*Borrelia* C6IgG1). Analysis was performed using the Stratec Gemini instrument (Stratec, Birkenfeld, Germany). Antibody levels were calculated in arbitrary units (AU) using the optical density (OD) values of the samples in the following equation:

AU = OD value/OD blank × 3

AU values > 2.10 were considered positive. Seroconversion was defined as a change from seronegative to seropositive or an increase in AU between the two samples (delta check) of at least 32.3% (sample 1 < 2.10) or 18.3% (sample 1 2.10–30 AU) or 9.5% (sample 1 > 30 AU), respectively, according to the manufacturer’s instructions (yet to be published). Participants who showed seroconversion, with or without clinical symptoms, were considered to have a current *Borrelia* infection. Seroprevalence was determined based on the number of *Borrelia* C6IgG1 positive serum samples collected at the time of inclusion.

Seroconversion and/or significant increase in antibody levels between the two samples were confirmed with a bead-based Luminex method (recomBead *Borrelia*-IgG 2.0, Mikrogen, Neuried, Germany), performed according to the manufacturer’s instructions.

### 2.9. Detection of TBEV-Specific IgM and IgG Antibodies

All serum samples were analyzed for TBEV IgM antibodies using a semi-quantitative point of care (POC) immune test (ReaScan, Reagena, Toivala, Finland) according to the manufacturer’s instructions. Samples showing >30 on the ReaScan reader were considered positive. Seroconversion was defined as a change from seronegative to seropositive or a fourfold increase in value between the two samples [30].

Serum samples 1 and 2 were analyzed in parallel with an ELISA detecting TBEV IgG antibodies (Euroimmun, Lübeck, Germany) according to the manufacturer’s instructions. Samples showing >22 RU/mL were considered positive. Seroconversion was defined as a change from seronegative to seropositive or an increase in RU/mL between the two samples of at least by a factor of two to four [30]. Seroprevalence was determined based on the number of TBEV IgG positive serum samples collected at the time of inclusion. 

### 2.10. Statistical Methods

The nonparametric Mann–Whitney U test and Kruskal–Wallis tests were used to compare numeric variables. Categorical variables were analyzed using Chi2, and Pearson was used for correlation analysis. A *p*-value ≤ 0.05 was considered statistically significant. The statistical analyses were conducted using IBM SPSS Statistics version 27.

## 3. Results

### 3.1. Tick Characteristics

In total, 425 ticks were collected: 100 at inclusion and 325 additional ticks during the two-month study period. Of all ticks, 307 were nymphs (72.2%), followed by 80 larvae (18.8%), 31 adult females (7.3%), and five adult males (1.2%). Two ticks (0.5%) were fragmented; therefore, their developmental stage could not be determined (Table 3). No significant differences were observed when the age or sex of the participant were compared with the developmental stage of the tick. All ticks were determined to be *I. ricinus*. Of the nymphs, 116 (38%) were estimated to have fed for ≤24 h, 129 (42%) for 25–47 h, and 44 (14%) for ≥48 h. Among the adult females, 14 (45%) had fed for ≤24 h, eight (26%) for 25–47 h, and one had fed for ≥48 h (3%). A significant difference (*p* = 0.01) was observed in the feeding times where the nymphs (4–100 h, median 27 h) had a longer feeding time than the adult females (6–54 h, median 27 h).

Due to the poor condition of some of the collected ticks, the feeding time could not be estimated for 18 nymphs and eight adult females. No significant differences were observed when the age or sex of the participant, or the developmental stage of ticks were compared with tick feeding time.

### 3.2. Prevalence of Borrelia and TBEV in Ticks

Of the 425 analyzed ticks, 84 (19.7%) contained *Borrelia* belonging to the *B.b.* sl. group. By sequencing the positive samples, *B. afzelii* was shown to be the most common species; 16 (19.1%), followed by nine *B. garinii* (10.7%), three *B. valaisiana* (3.6%), and two *B. burgdorferi* sensu stricto (2.4%). There was a single finding of *B. spielmanii* (1.2%). Lastly, 53 samples (63.1%) could not be typed to species level based on the obtained sequences (Table 3). All the obtained sequences used for species determination can be found in Supplementary File S1. Of all ticks, nymphs were the most common developmental stage to contain *Borrelia* (16.7%), followed by adult females (2.8%) (Table 3). However, 39% of all 31 collected adult females contained *Borrelia*, and 23% of all 307 collected nymphs contained *Borrelia*. Neither adult males nor larvae contained *Borrelia* (Table 3). None of the ticks contained *B. miyamotoi* or TBEV. 

### 3.3. Study Cohort

In total 109 participants were included from August 2018 to June 2022. In total, nine participants were excluded (8.2%): five due to medical treatment, one due to an undetectable tick bite site, two due to illness at the time of biopsy, and one with insufficient sample material. Among the 100 enrolled participants, 59 (59%) were female. The median age was 65 years (29–87 years). There was no significant difference in the age of males and females. Of the included, 62 (62%) were without chronic medical conditions and 52 (52%) had no ongoing medical treatments. 

Most of the participants reported being tick-bitten on the main island of Aland (85%), and the rest were bitten in the archipelago (15%). Reported tick bite locations were: 21 (21%) on the torso, 54 (54%) on legs, and 25 (25%) on the arms. The sites were not significantly different between the sexes or dependent on the age of the participants. Earlier tick bites in the same season were reported by 81 (81%) participants, of whom 54 (54%) had noticed more than five tick bites in the same season. At the eight-week follow-up, 72 (72%) reported additional tick bites during the study period, whereof 60 (60/72, 83%) did include the additional ticks for analysis. The mean additional tick per participant was 3.2. 

As a result of the TBE vaccination program, 86 (86%) of the participants reported complete vaccination status including three doses. Booster doses were not registered due to lack of information.

All participants completed the follow-up visit after eight weeks, and 87 (87%) participated in the four-month follow-up by phone. Thirteen (13%) did not participate due to different reasons; twelve could not be reached and one was deceased.

### 3.4. Borrelia Antibody Analysis

The seroprevalence of *Borrelia* C6IgG1 was 57% (26 males, 31 females). Previous LB was reported by 44 (44%) participants, of whom 32 (32/44, 72%) were positive for *Borrelia* C6IgG1 at inclusion.

In total 14 (14%) seroconverted or had increased *Borrelia* C6IgG1 levels at the eight-week follow-up (7 males, 7 females). Nine (9/14, 64%) of the seroconverted were asymptomatic. Ten (10/14, 71%) were bitten by a *Borrelia*-positive tick, of whom three contained *B. garinii*, two *B. afzelii*, one *B. valaisiana*, and four were untypeable (Table S1). The estimated tick feeding time for the seroconverted participants was 29 h (<24–59 h) including inclusion and additional ticks, respectively.

### 3.5. TBEV Antibody Analysis

**Figure 2:**
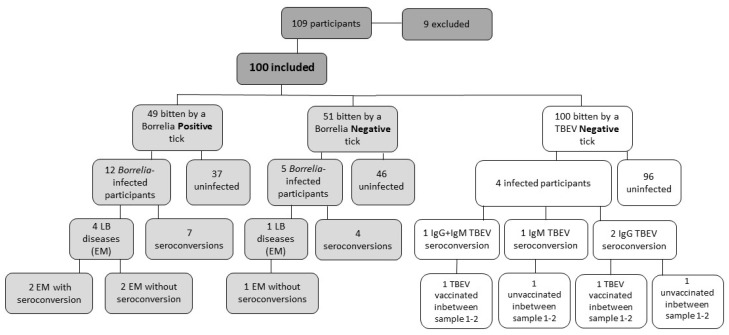
Flow chart of the study participants and their corresponding tick-pathogen content, antibody response, and clinical diagnosis. All ticks (collected at inclusion and additionally detached ticks during the study period) are included in the calculation of *Borrelia* and tick-borne encephalitis virus (TBEV) positive/negative ticks. The definition of infected participants is seroconversion and/or clinical diagnosis of Lyme borreliosis (LB) and tick-borne encephalitis eight weeks and four months after inclusion. Abbreviations: EM; *erythema migrans*.

At inclusion, the seroprevalence of TBEV IgM was 2% and of TBEV IgG 52%. In total, four (4%) participants seroconverted during the first eight weeks, two (2/4, 50%) had increased IgG levels, one (1/4, 25%) had increased IgM and one (1/4, 25%) had increased IgG and IgM levels, respectively. (Table S1, Figure 2). All of the participants developing TBEV antibodies had been vaccinated with at least three doses prior to the study. 

Five (5%) reported previous TBE of which four (4/5, 80%) had positive IgG antibodies at the time of inclusion.

Among the 86 TBE vaccinated (at least three doses) participants, 52 (52/86, 60.4%) had IgG antibodies at inclusion. Between samples one and two, three (3%) participants had reported a booster vaccination of TBE, whereof two (2/3, 67%) showed increased IgG antibody levels.

### 3.6. Symptoms, Clinical Manifestations, and Seroconversion

Five (5%) participants developed *erythema migrans* (EM), of whom two (2/5, 40%) seroconverted in *Borrelia* C6IgG1 antibodies. One (1%) participant was treated with antibiotics for an unspecific rash, considered unlikely to be EM. No other manifestations of LB were observed or diagnosed. Among the EM cases, four (4/5, 80%) were bitten by a *Borrelia*-positive tick. The ticks contained *B. garinii*, *B. afzelii*, and two untypeable *Borrelia* species, respectively (Figure 2, Table S1). The estimated tick feeding time for the ticks detached from the participants who developed EM was 32 h (<24–65 h), including inclusion and additional ticks, respectively.

Six (6%) participants were prescribed antibiotics during the first eight weeks, of whom four (4/6, 66.7%) were due to EM and two (2/6, 28.6%) were due to other medical reasons. These two participants did not seroconvert during the first eight weeks. 

Forty-nine (49%) were bitten by a *Borrelia*-positive tick. Four of them developed EM (4/49, 8.1%) and seven (7/49, 14%) seroconverted without any clinical diagnosis. Fifty-one (51%) were bitten by a *Borrelia*-negative tick, however, four of them seroconverted (4/51, 7.8%) (Figure 2).

Eleven out of the 14 (11/14, 79%) who had seroconverted in *Borrelia* C6IgG1 were asymptomatic during the first eight weeks. Three (3/14, 21%) showed symptoms that had disappeared at the final follow-up. Three (3/14, 21%) participants reported symptoms after eight weeks. Symptoms are presented in Supplementary Table S1. The tick feeding time did not significantly affect the risk of seroconversion or development of EM. 

Of those seroconverted in TBEV IgG and IgM, two (2/4, 50%) did report TBE vaccination during the first eight weeks. The other two participants did not report any flavivirus vaccination during the first eight weeks. The only participant with increased TBEV IgG antibodies without recent vaccination showed no clinical symptoms associated with TBE (Figure 2, Table S1). Another participant with increased TBEV IgM antibodies, without recent vaccination, had clinical symptoms such as chills, fever, fatigue, nausea, loss of appetite, and headache during the first eight weeks. The headache and fatigue persisted after four months (Table S1). This participant, however, did not seek medical care.

## 4. Discussion

The AxBioTick study is an ongoing study conducted on the Aland Islands. In this paper, we present the study design, tick characteristics, the prevalence of *Borrelia* spp. and TBEV in ticks that have bitten humans, and the antibody responses. Furthermore, we aimed to investigate if tick characteristics and antibody responses affect the clinical response in tick-bitten individuals. 

As expected, the only tick species detected to feed on humans was *I. ricinus*. The method used in this paper can distinguish between *I. ricinus* and *I. persulcatus*. These closely related species are both vectors for *Borrelia* spp. and TBEV, among other TBPs. Although *I. ricinus* is the main vector of TBPs in northern Europe, several studies report findings of *I. persulcatus* among questing ticks in Sweden and the mainland Finland [17,22,31]. The prevalence of *I. persulcatus* ticks is of particular interest to monitor. They are vectors for the Siberian and Far-Eastern subtypes of TBEV, the latter associated with more severe disease. In addition, their geographical distribution is expanding as a result of ongoing climate changes and bird migration. 

We found that 20% of the analyzed ticks contained *Borrelia* spp., among which *B. afzelii* was predominant, followed by *B. garinii*. There were also findings of *B. valaisiana*, *B. burgdorferi* sensu stricto, and *B. spielmanii*, but no findings of *B. miyamotoi*. These findings are in line with a previous study by Wilhelmsson et al. [5], where 23% of all ticks detached from participants from the Aland Islands contained *Borrelia* spp. except for two findings of *B. miyamotoi* (1%) [5]. Similarly, 19% of ticks detached from Swedish participants contained *Borrelia* spp. and merely a single finding of a *B. miyamotoi*-like species [27]. Studies from Sweden and mainland Finland report a prevalence of *B. miyamotoi* in ticks ranging from 0.5 to 4% [17,22,27]. We observed a higher prevalence of *Borrelia* spp. in the adult females compared to nymphs, which is likely related to the higher number of blood meals ingested by the adult tick. There were no findings of *Borrelia* spp. in the larvae, which reinforces the theory that transovarial transmission of *Borrelia* spp. is rare [32].

The risk of transmission of *Borrelia* spirochetes to humans has been noticed to increase with the tick feeding time [33,34]. An increased transmission rate has been observed if the tick has fed for >24 h [34]. The feeding time, in turn, seems to increase with the age of the tick-bitten person and in males [35]. However, in our study, we did not observe that the feeding time was affected by any of these factors. The median feeding time of the ticks from participants who developed EM was 32 h, further strengthening the evidence that delayed removal of a tick may increase the risk of infection. The nymphs had a significantly longer feeding time than the adult females. This is contrary to what was described in the TBD STING-study, where the discrepancy was discussed to be due possibly to increased awareness of the participants [35]. In both the TBD STING-study and the AxBioTick study, the coxal and the scutal index are applied to estimate the feeding time of the ticks giving more accurate estimations [21]. Thus, the discrepancy may depend on the size of the cohort as the TBD STING-study included 20 times more ticks [35]. The majority of the ticks in this study were nymphs, as in the TBD STING-study. However, in this study almost five times as many larvae were collected [35]. Seasonal differences might contribute to differences in the proportions of tick developmental stages. Due to an abundance of ticks, there is an increased awareness of tick-borne diseases and ticks in the population on the Aland Islands resulting in a behavior of carefully inspecting oneself after exposure and removing the tick immediately after being noticed. Although the inhabitants do tend to have a high number of tick bites per season. All these circumstances could lead to a higher proportion of larvae collected in this study. 

In total, 17 participants showed signs of a current *Borrelia* infection. Increased antibodies and seroconversions of the *Borrelia* C6IgG1 antibodies were noted in 14% of the participants, and in total five participants were diagnosed with EM. In the TBD STING-study, 3.5% of the participants seroconverted, of whom 3% were asymptomatic and 2% developed clinical manifestations of LB [1]. Interestingly, the number of infected participants was higher in this study. Although the cohort size was smaller, the median tick feeding time of this group was >24 h, leading to a higher risk of transmission of *Borrelia* spirochetes and thereby infection [34]. Even though a high number of participants was infected, not all were bitten by a *Borrelia*-positive tick. The infection could have originated from earlier tick bites or additional tick bites during the study, which were not noticed and/or collected.

None of the analyzed ticks contained TBEV. Two participants had increased TBEV IgM or TBEV IgG antibodies without recent vaccination to TBEV or other flaviviruses. These participants may have been bitten by TBEV-infected tick(s) before the study, by a tick that was not noticed and/or collected, or possibly through food-borne transmission which explains approximately 1% of all TBE cases in Europe [36]. As with all laboratory testing, there are limitations in the assays. The TBEV antibody kits used in this study could have cross-reactivity against all different flaviviruses, although we consider it unlikely since the overall specificity of the IgM assay is 97.7% and IgG is 100% according to the manufacturers [37]. With an estimated 99% confidence interval of 22.3–37.7 for the IgM cut-off-value, the assay may present some false positive samples in concentrations near the cut-off, leading to misdiagnosis. 

A notable strength of our study is the thorough study design. We managed to collect blood samples, ticks, and skin biopsies from a cohort with an exceptionally high participation rate. Despite the many samplings, questionnaires, and follow-ups required, only 13% did not complete the full study. A few limitations remain, as well, to be mentioned. The enrolled participants had a somewhat higher median age (65 years) compared to the average age of the population on the Aland Islands (44 years, 2021) [38]. The opening hours of the inclusion center overlap with traditional working hours, which could have led to more participants of retirement age being enrolled. This phenomenon has been seen in other studies and the overall participation rate in scientific studies among older persons is known to be higher [20,39]. Further, the species of many *Borrelia*-positive ticks could not be determined. Several steps in the analysis process could have been faulty, such as extraction and amplification of PCR products, storage, other species of the relapsing fever group of *Borrelia*, few *Borrelia* copies as well as co-infections with several *Borrelia* species. The cohort size was chosen to contain the first 100 participants which might cause some bias, albeit it gives important insights.

Some of our findings differ from what was expected from the TBD STING-study, which is to be considered a precursor of the AxBioTick study. As 16 years have passed since the TBD STING-study was initiated, it is not unlikely that some results will differ. It will be of great interest to further investigate the participants that are yet to be included to delineate if the changes are persistent. 

## 5. Conclusions

In this first part of the AxBioTick study, we found a high seroprevalence of both *Borrelia* and TBEV antibodies. Few participants had clinical manifestations of LB, despite a high number of seroconversions. Even though the Aland Island is endemic for TBE, TBEV was not found in any ticks and there were no confirmed clinical cases. The prevalence and diversity of *Borrelia* were found to be similar to findings in Sweden and mainland Finland. The AxBioTick study continues to investigate more participants and ticks for co-infections, and to characterize the dermal immune response following a tick bite. 

## Figures and Tables

**Table 1:** Target genes, primers, and probes used for molecular identification of tick species.

Target Gene	Primer/Probe Names	Sequence (5′ to 3′)	Product Length (bp)	Reference
ITS2	IXO-I2-F4	TCTCGTGGCGTTGATTTGC	95	[22]
	IXO-I2-R4	CTGACGGAAGGCTACGACG		
	Ipe-I2-P4	FAM-TGCGTGGAAAGAAAACGA G-BHQ1		
	Ipe-I2-4	VIC-TGCTCGAAGGAGAGAACG A-BHQ1		

Abbreviations: FAM, 6-carboxyfluorescein; BHQ, black hole quencher; VIC, 6-carboxy-rhodamine.

**Table 2:** Target genes, primers, and probes used for detection and species determination of *Borrelia* spp. and TBEV.

Pathogen	Target Gene	Primer/ Probe Names	Sequence (5′ to 3′)	Product Size (bp)	References
*Borrelia* spp.	16S rRNA	Borrelia-F	GCTGAGTCACGAAAGCGTAG	116	[24]
		Borrelia-R	CACTTAACACGTTAGCTTCGG TATACTAAC		
		Borrelia-P	FAM-CGCTGTAAA CGA TGC ACA CTT GGT-MGB		
*B. miyamotoi*	*flab*	Bm_F	AGAAGGTGCTCAAGCAG	115	[25]
		Bm_R	TCGATCTTTGAAAGTGACATAT		
		Bm_P	FAM-AGCACAACAGGAGGGAGTTCA AGC-BHQ1		
*B. burgdorferi* s.l.	5S–23S rRNA IGS	B5S-23S_F	CTGCGAGTTCGCGGGAGA	225–266	[26]
		B5S-23S_R	TCCTAGGCATTCACCATA		
		B5S-23S_Fn	GAGTTCGCGGGAGAGTAA	131	[27]
		B5S-23S_Rn	TAGGCATTCACCATAGACTCTT		
TBEV	11,054–11,121 ^a^	F-TBE 1	GGGCGGTTCTTGTTCTCC	68	[28]
		R-TBE 1	ACACATCACCTCCTTGTCAGACT		
		TBE-probe-WT	FAM-TGAGCCACCATCACCCAG ACACA-BHQ1		
	1329–1416 ^a^	TBEE-F6	GGCTGTGAGGCAAAAAAGAA	88	[29]
		TBEE-R2	TCCCGTGTGTGGTTCGACTT		
		TBEE-P4	HEX-AGCCACAGGACATGTGTACGACGCC-BHQ1		

^a^ = Genome region of TBEV strain *Neudoerfl* (U27495) which is present in all three subtypes of TBEV. Abbreviations: FAM, 6-carboxyfluorescein; MGB, minor groove binder; BHQ, black hole quencher; HEX, 5(6)-carboxyfluorescein.

**Table 3:** Developmental stage and prevalence of *Borrelia* in the collected ticks, and the distribution of *Borrelia* species in the *Borrelia* positive ticks.

Developmental Stage	Examined Ticks n (%)	*Borrelia* Positive Ticks n (%)	*Borrelia* Species Determined by Nucleotide Sequencing; n (%)					
			*B. a.*	*B. g.*	*B. v.*	*B.b.* ss.	*B. s.*	Unknown
Adult female	31 (7.3)	12 (2.8)	2 (2.4)	3 (3.6)	-	1 (1.2)	-	6 (7.1)
Adult male	5 (1.2)	-	-	-	-	-	-	-
Nymph	307 (72.2)	71 (16.7)	14 (16.7)	6 (7.1)	3 (3.5)	1 (1.2)	1 (1.2)	46 (54.8)
Larvae	80 (18.8)	-	-	-	-	-	-	-
Untypeable	2 (0.5)	1 (0.2)	-	-	-	-	-	1 (1.2)
Total	425 (100)	84 (19.7)	16 (19.1)	9 (10.7)	3 (3.5)	2 (2.4)	1 (1.2)	53 (63.1)

Abbreviations: *B. a.*:* Borrelia afzelii*; *B. g.*: *Borrelia garinii*; *B. v.*: *Borrelia valaisiana*; *B.b. ss.*: *Borrelia burgdorferi* sensu stricto; *B. s.*: *Borrelia spielmanii.*

## Data Availability

The data supporting the conclusions of this article are included within the article. Raw data can be shared with researchers upon request.

## Supplementary Materials

The following supporting information can be downloaded at: https://www.mdpi.com/article/10.3390/microorganisms11051100/s1, Supplementary File S1: Aligned *Borrelia* nucleotide sequences based on PCR-products.; Supplementary Table S1: *Borrelia* and Tick-borne encephalitis virus (TBEV) infected participants, eight-weeks (+/− 2 weeks) after tick-bite.

## References

[1] Wilhelmsson P., Fryland L., Lindblom P., Sjöwall J., Ahlm C., Berglund J., Haglund M., Henningsson A.J., Nolskog P., Nordberg M. (2016). A Prospective Study on the Incidence of Borrelia Burgdorferi Sensu Lato Infection after a Tick Bite in Sweden and on the Åland Islands, Finland (2008–2009). Ticks Tick-Borne Dis..

[2] Lindblom P., Wilhelmsson P., Fryland L., Matussek A., Haglund M., Sjöwall J., Vene S., Nyman D., Forsberg P., Lindgren P.-E. (2014). Factors Determining Immunological Response to Vaccination against Tick-Borne Encephalitis Virus in Older Individuals. PLoS ONE.

[3] Taulukko. Puutiaisaivotulehduksen Esiintyvyys ja Rokotussuositukset Tartuntapaikkakunnittain-THL.

[4] Uppföljningen och förekomsten av borrelios i Finland—THL. https://thl.fi/sv/web/infektionssjukdomar-och-vaccinationer/sjukdomar-och-bekampning/sjukdomar-och-sjukdomsalstrare-a-o/borrelia/uppfoljningen-och-forekomsten-av-borrelios-i-finland.

[5] Wilhelmsson P., Lindblom P., Fryland L., Ernerudh J., Forsberg P., Lindgren P.-E. (2013). Prevalence, Diversity, and Load of Borrelia Species in Ticks That Have Fed on Humans in Regions of Sweden and Åland Islands, Finland with Different Lyme Borreliosis Incidences. PLoS ONE.

[6] Houžvičková A., Dorko E., Rimárová K., Diabelková J., Drabiščák E. (2022). Seroprevalence of Borrelia IgG Antibodies among Individuals from Eastern Slovakia. Cent. Eur. J. Public Health.

[7] Barreiro-Hurlé L., Melón-García S., Seco-Bernal C., Muñoz-Turrillas C., Rodríguez-Pérez M. (2020). Seroprevalence of Lyme Disease in Southwest Asturias. Enfermedades Infecc. Microbiol. Clin. Engl. Ed..

[8] Munro H., Mavin S., Duffy K., Evans R., Jarvis L.M. (2015). Seroprevalence of Lyme Borreliosis in Scottish Blood Donors: Letter to the Editor. Transfus. Med..

[9] Wilking H., Fingerle V., Klier C., Thamm M., Stark K. (2015). Antibodies against Borrelia Burgdorferi Sensu Lato among Adults, Germany, 2008–2011. Emerg. Infect. Dis..

[10] Hristea A., Hristescu S., Ciufecu C., Vasile A. (2001). Seroprevalence of Borrelia Burgdorferi in Romania. Eur. J. Epidemiol..

[11] Svensson J., Christiansen C.B., Persson K.E.M. (2021). A Serosurvey of Tick-Borne Encephalitis Virus in Sweden: Different Populations and Geographical Locations. Vector-Borne Zoonotic Dis..

[12] Hjetland R., Henningsson A.J., Vainio K., Dudman S.G., Grude N., Ulvestad E. (2015). Seroprevalence of Antibodies to Tick-Borne Encephalitis Virus and Anaplasma Phagocytophilum in Healthy Adults from Western Norway. Infect. Dis..

[13] Nyman D. (Borrelia Research Group of the Aland Islands, Mariehamn, the Aland Islands, Finland). TBEV IgG Antibodies in Healthy Blood Donors from 1995 and 2018.

[14] Steere A.C., Coburn J., Glickstein L. (2004). The Emergence of Lyme Disease. J. Clin. Investig..

[15] Marques A.R., Strle F., Wormser G.P. (2021). Comparison of Lyme Disease in the United States and Europe. Emerg. Infect. Dis..

[16] Cardenas-de la Garza J.A., De la Cruz-Valadez E., Ocampo-Candiani J., Welsh O. (2019). Clinical Spectrum of Lyme Disease. Eur. J. Clin. Microbiol. Infect. Dis..

[17] Sormunen J.J., Andersson T., Aspi J., Bäck J., Cederberg T., Haavisto N., Halonen H., Hänninen J., Inkinen J., Kulha N. (2020). Monitoring of Ticks and Tick-Borne Pathogens through a Nationwide Research Station Network in Finland. Ticks Tick-Borne Dis..

[18] Riccardi N., Antonello R.M., Luzzati R., Zajkowska J., Di Bella S., Giacobbe D.R. (2019). Tick-Borne Encephalitis in Europe: A Brief Update on Epidemiology, Diagnosis, Prevention, and Treatment. Eur. J. Intern. Med..

[19] Oker-Blom N., Kääriäinen L., Brummer-Korvenkontio M., Weckström P. (1962). Isolation and Occurrence of the Viruses of the Tick-Borne Encephalitis Complex in Finland. Biol. Viruses Tick-Borne Enceph. Complex.

[20] Fryland L., Wilhelmsson P., Lindgren P.-E., Nyman D., Ekerfelt C., Forsberg P. (2011). Low Risk of Developing Borrelia Burgdorferi Infection in the South-East of Sweden after Being Bitten by a Borrelia Burgdorferi-Infected Tick. Int. J. Infect. Dis..

[21] Gray J., Stanek G., Kundi M., Kocianova E. (2005). Dimensions of Engorging Ixodes Ricinus as a Measure of Feeding Duration. Int. J. Med. Microbiol..

[22] Sormunen J.J., Penttinen R., Klemola T., Hänninen J., Vuorinen I., Laaksonen M., Sääksjärvi I.E., Ruohomäki K., Vesterinen E.J. (2016). Tick-Borne Bacterial Pathogens in Southwestern Finland. Parasit. Vectors.

[23] Halos L., Jamal T., Vial L., Maillard R., Suau A., Le Menach A., Boulouis H.-J., Vayssier-Taussat M. (2004). Determination of an Efficient and Reliable Method for DNA Extraction from Ticks. Vet. Res..

[24] Gyllemark P., Wilhelmsson P., Elm C., Hoornstra D., Hovius J.W., Johansson M., Tjernberg I., Lindgren P.-E., Henningsson A.J., Sjöwall J. (2021). Are Other Tick-Borne Infections Overlooked in Patients Investigated for Lyme Neuroborreliosis? A Large Retrospective Study from South-Eastern Sweden. Ticks Tick-Borne Dis..

[25] Hovius J.W.R., de Wever B., Sohne M., Brouwer M.C., Coumou J., Wagemakers A., Oei A., Knol H., Narasimhan S., Hodiamont C.J. (2013). A Case of Meningoencephalitis by the Relapsing Fever Spirochaete Borrelia Miyamotoi in Europe. Lancet.

[26] Postic D., Assous M.V., Grimont P.A.D., Baranton’ G. (1994). Diversity of Borrelia Burgdorferi Sensu Lato Evidenced by Restriction Fragment Length Polymorphism of Njf (5S)-Rrl (23s) Intergenic Spacer Amplicons. Int. J. Syst. Bacteriol..

[27] Wilhelmsson P., Fryland L., Börjesson S., Nordgren J., Bergström S., Ernerudh J., Forsberg P., Lindgren P.-E. (2010). Prevalence and Diversity of Borrelia Species in Ticks That Have Bitten Humans in Sweden. J. Clin. Microbiol..

[28] Schwaiger M., Cassinotti P. (2003). Development of a Quantitative Real-Time RT-PCR Assay with Internal Control for the Laboratory Detection of Tick Borne Encephalitis Virus (TBEV) RNA. J. Clin. Virol..

[29] Gäumann R., Mühlemann K., Strasser M., Beuret C.M. (2010). High-Throughput Procedure for Tick Surveys of Tick-Borne Encephalitis Virus and Its Application in a National Surveillance Study in Switzerland. Appl. Environ. Microbiol..

[30] Wellinghausen N., Abele-Horn M., Donoso Mantke O., Enders M., Fingerle V., Gärtner B., Hagedorn J., Rabenau H.F., Reiter-Owona I., Titelknot K. (2018). MiQ, Immunological Methods for the Detection of Infectious Diseases.

[31] Jaenson T.G.T., Wilhelmsson P. (2019). First Records of Tick-Borne Pathogens in Populations of the Taiga Tick Ixodes Persulcatus in Sweden. Parasit. Vectors.

[32] Tilly K., Rosa P.A., Stewart P.E. (2008). Biology of Infection with Borrelia Burgdorferi. Infect. Dis. Clin. N. Am..

[33] Sood S.K., Salzman M.B., Johnson B.J.B., Happ C.M., Feig K., Carmody L., Rubin L.G., Hilton E., Piesman J. (1997). Duration of Tick Attachment as a Predictor of the Risk of Lyme Disease in an Area in Which Lyme Disease Is Endemic. J. Infect. Dis..

[34] Cook M. (2014). Lyme Borreliosis: A Review of Data on Transmission Time after Tick Attachment. Int. J. Gen. Med..

[35] Wilhelmsson P., Lindblom P., Fryland L., Nyman D., Jaenson T.G., Forsberg P., Lindgren P.-E. (2013). Ixodes Ricinus Ticks Removed from Humans in Northern Europe: Seasonal Pattern of Infestation, Attachment Sites and Duration of Feeding. Parasit. Vectors.

[36] Buczek A.M., Buczek W., Buczek A., Wysokińska-Miszczuk J. (2022). Food-Borne Transmission of Tick-Borne Encephalitis Virus—Spread, Consequences, and Prophylaxis. Int. J. Environ. Res. Public. Health.

[37] Albinsson B., Jääskeläinen A.E., Värv K., Jelovšek M., GeurtsvanKessel C., Vene S., Järhult J.D., Reusken C., Golovljova I., Avšič-Županc T. (2020). Multi-Laboratory Evaluation of ReaScan TBE IgM Rapid Test, 2016 to 2017. Eurosurveillance.

[38] Befolkningens åldersstruktur 31.12.2021|Ålands statistik- och utredningsbyrå. https://www.asub.ax/sv/statistik/befolkningens-aldersstruktur-31122021.

[39] Galea S., Tracy M. (2007). Participation Rates in Epidemiologic Studies. Ann. Epidemiol..

