# Supplementary material for: The AxBioTick Study: Borrelia Species and Tick-Borne Encephalitis Virus in Ticks, and Clinical Responses in Tick-Bitten Individuals on the Aland Islands, Finland
Source: Microorganisms. 2023 Apr 22;11(5):1100. doi: 10.3390/microorganisms11051100 (PMC10223457; doi:10.3390/microorganisms11051100)
Supplement: Supplementary file 1 [file microorganisms-11-01100-s001.zip › Supplementary File S1.pdf]

Supplementary File S1. Aligned *Borrelia* nucleotide sequences based on PCR-products.

The identifier line, which begins with '>', gives the name of the **sample ID_*Borrelia* species**

>AX2-2-2 ***B. garinii***
GAGTAAGTTATTGCCAGGCGTTTTTATTTTATACTTTAAACATTGATTTTATTTTTTATGTT
TTTAGATAGTTTCATGTTTTTGAATGTTTTATTCAAATAATATAAAAAATAAAATATATA
TTGACATGGATTAAACAAAGATATATATTATTCTATGTTGTATAAACAAATTGGCAAAA
TAGAGATGGAAGATAAAAATATGGTCAAAGTAATAAGAGTCTATGGTGAATGCCTA

>AX4 ***B. burgdorferi***
GAGTTCGCGGGAGAGTAAGTTATTGCCAGGGTTTTTATTTTTATACTTTAAACTTTGATTT
TATTTTTATGTTTTTTAAATATTGGTGTTTTTGAATGTGTTGTTTAAATAACATAAAAAAT
AAAATATATATATTGACATGCATTAAACAAAGATATATATTATTTTATGTTGTATAAATA
AATTGGCAAAATAGAGATGGA

>AX5-2-5 ***B. garinii***
GGGAGAGTAAGTTATTGCCAGGGTTTTTCTTTTATACTTTAAACATTGATTTTATTTTTTA
TGTTTTTAGATGTTTATATTATTTGAATGTTTTATTCAAATAATATAAAAAATAAAATATA
TATATTGACATGGATTAAACAAAGATATATATTATTCTATGTTGTATAAACAAATTGGC
AAAATAGAGATGGAAGATAAAAATATGGTCAAAGTAATAAGAGTCTATGGTGAATGCC
TAG

>AX6-2-3 ***B. spielmanii***
GAGTTCGCGGGAGAGTAAGTTATTGCCAGGGTTTTTATTTTTTTTAATGTTTATGTTATTT
GAATAAGCCATTCAAATAACATAAAAAAGAAAATAGATATTGACATGGATTAAACAA
AGATATATATTATTCTATGTTGCATAAACAAATTGGCAAAGTAGAGATGGAAGAT
AAAAATATGGTCAAAGTAATAAGAGTCTATGG

>AX6-2-6 ***B. garinii***
AGTTCGCGGGGAGAGTAAGTTATTGCCAGGGTTTTTATTTTATACTTTAAACATTGATTTT
ATTTTTTATGTTTTTAGATGTTTATGTTTTTGAATGTTTTATTCAAATAATATAAAAAATA
AAATATATATTGACATGGATTAAACAAAGATATATATTATTCTATGTTGTATAAACAAA
TTGGCAAAATAGAGATGGAAGATAAAAATATGGTCAAAGTAATAAGAGTCTATGGTGA
ATGCCTAG

>AX7-2-1 ***B. burgdorferi*** **sensu stricto**
GAGTTCGCGGGGAGAGTAAGTTATTGCCAGGGTTTTTATTTTTATACTTTAAACTTTGATT
TTATTTTTATGTTTTTTAAATATTGGTGTTTTTGAATGTGTTGTTTAAATAACATAAAAAA
TAAAATATATATATTGACATGCATTAAACAAAGATATATATTATTTTATGTTGTATAAAT
AAATTGGCAAAATAGAGATGGAAGATAAAAATATGGTCAAAGTAATAAGAGTCTATG
GT

>AX12-2-9 ***B. afzelii***
GAGTTCGCGGGAGAGTAAGTTATTGCCAGGGTTTTTATTTTATACTTTAAACCTTGAATT
TATTTTTTAAATGTTTATATTATTTGAATAAAACATTCAAATAATATAAAAAATAATATA
TATATTGACATGGATTAAACAAAGATATATATTATTCTATGTTGTATGAACAAATTGGC
AAAATAGAGATGAAGATAAAAATATGGTCAAAGTAATAAGAGTCTATGGTGAATGCCT
A

>AX12-2-10 ***B. afzelii***
GAGTTCGCGGGGAGAGTAAGTTATTGCCAGGGTTTTTATTTTATACTTTAAACCTTGAAT
TTATTTTTTAAATGTTTATATTATTTGAATAAAACATTCAAATAATATAAAAAATAATAT
ATATATTGACATGGATTAAACAAAGATATATATTATTCTATGTTGTATGAACAAATTGG
CAAAATAGAGATGAAGATAAAAATATGGTCAAAGTAATAAGAGTCTAT

>AX12-2-11 ***B. valaisiana***
GTTCGCGGGGAGAGTAAGTTATTGCCAGGGTTTTTATTTTGTACTTTAAACCTTAAATTT
ATTTTTTATATTTTTTTAATGTTCATGTTTTTGAATGTTTTATTCAAATAATGTAAAAAATA
AAATAGATATTGACATGGATTGAACAAAAGATATATATTATTTTATGTTGCATAAACAA
ATTGGCAAAATAGAGATGGAAGATAAAAATATGGTCAAAGTAATAAGAGTCTATGGT

>AX23 ***B. valaisiana***
GTTCGCGGGAGAGTAAGTTATTGCCAGGGTTTTTATTTTGTACTTTAAACCTTAAATTTAT
TTTTTATATTTTTTTAATGTTCATGTTTTTGAATGTTTTATTCAAATAATGTAAAAAATAA
AATAGATATTGACATGGATTGAACAAAAGATATATATTATTTTATGTTGCATAAACAAA
TTGGCAAAATAGAGATGGAAGATAAAAATATGGTCAAAGTAATAAGAGTCTATGGTGA
ATGCCTA

>AX23-2-7 ***B. garinii***
GAGTAAGTTATTGCCAGGCGTTTTTATTTTATACTTTAAACATTGATTTTATTTTTTATGTT
TTTAGATATTCATGTTTTTGAATGTTTTATTCGAATAATATAAAAAATAAAATATATATTT
TGACATGGATTAAACAAAGATATATATTATTCTATGTTGTATAAACAAATTGGCAAAAT
AGAGATGGAAGATAAAAATATGGTCAAAGTAATAAGA

>AX23-2-8 ***B. afzelii***
GAGTTCGCGGGAGAGTAAGTTATTGCCAGGGTTTTTATTTTATACTTTAAACCTTGAATT
TATTTTTTAAATGTTTATATTATTTGAATAAAACATTCAAATAATATAAAAAATAATATA
TATATTGACATGGATTAAACAAAGATATATATTATTCTATGTTGTATAAACAAATTGGC
AAAATAGAGATGAAGATAAAAATATGGTCAAAGTAATAAGAGTCTATGGTGAATGCC

>AX26 ***B. afzelii***
GAGTTCGCGGGAGAGTAAGTTATTGCCAGGGTTTTTATTTTATACTTTAAACCTTGAATT
TATTTTTTAAATGTTTATATTATTTGAATAAAACATTCAAATAATATAAAAAATAATATA
TATATTGACATGGATTAAACAAAGATATATATTATTTTATGTTGTATAAACAAATTGGCA
AAATAGAGATGGAAGATAAAAATATG

>AX28 ***B. garinii***
GAGTAAGTTATTGCCAGGGTTTTTATTTTATACTTTAAACATTGATTTTATTTTTTATGTTT
TTAGATGTTCATGTTTTTGAATGTTTTATTCGAATAATATAAAAAATAAAATATATATTG
ACATGGATTAAACAAAGATATATATTATTCTATGTTGTATAAACAAATTGGCAAAATAG
AGATGGAAGATAAAAATATGGTCAAAGTAATAAGAGTCTATGGTGAATGCCTA

>AXF29-2-1 ***B. afzelii***
GAGTTCGCGGGGAGAGTAAGTTATTGCCAGGGTTTTTATTTTATACTTTAAACCTTGAAT
TTATTTTTTAAATGTTTATATTATTTGAATAAAACATTCAAATAATATAAAAAATAATAT
ATATATTGACATGGATTAAACAAAGATATATATTATTCTATGTTGTATAATACAAATTGG
CAAAATAGAGATGAAGATAAAAATATGGTCAAAGTAATAAGAGTCTATGGTGAATGCC

>AX30-2-3 ***B. afzelii***
GAGTTCGCGGGGAGAGTAAGTTATTGCCAGGGTTTTTATTTTATACTTTAAATCTTGAAT
TTATTTTTTAAATGTTTATATTATTTGAATAAAACATTCAAATAATATAAAAAATAATAT
ATATATTGACATGGATTAAACAAAGATATATATTATTCTATGTTGTATAAACAAATTGG
CAAAATAGAGATGGAAGATAAAATATGGTCAAAGTAATAAGAGTCTATGGT

>AX33 ***B. afzelii***
GAGTTCGCGGGAGAGTAAGTTATTGCCAGGGTTTTTATTTTATACTTTAAACCTTGAATT
TATTTTTTAAATGTTTATATTATTTGAATAAAACATTCAAATAATATAAAAAATAATATA
TATATTGACATGGATTAAACAAAGATATATATTATTCTATGTTGTATGAACAAATTGGC
AAAATAGAGATGGAAGATAAAAATATGGTCAAAGTAATAAGAGTCTATGGTGAATGCC
TA

>AX35 ***B. afzelii***
GAGTTCGCGGGAGAGTAAGTTATTGCCAGGGTTTTTATTTTATACTTTAAATCTTGAATTT
ATTTTTTAAATGTTTATATTATTTGAATGTTTTATTCAAATAATATAAAAAATAATATATA
TATTGACATGGATTAAACAAAGATATATATTATTCTATGTTGTATTAACAAATTGGCAA
AATAGAGATGGAAGATAAAAATATGGTCAAAGTAATAAGAGTCTATGGTGAATGCCTA

>AX39 ***B. afzelii***
CGAGTTCGCGGGAGAGTAAGTTATTGCCAGGGTTTTTATTTTATACTTTAAATCTTGAAT
TTATTTTTTAAATGTTTATATTATTTGAATGTTTTATTCAAATAATATAAAAAATAATATA
TATATTGACATGGATTAAACAAAGATATATATTATTCTATGTTGTATAAACAAATTGGC
AAAATAGAGATGGAAGATAAAAATATGGTCAAAGTAATAAGAGTCTATGGTGAATGCC
TA

>AX53 ***B. garinii***
GAGTTCGCGGGAGAGTAAGTTATTGCCAGGGTTTTTATTTTATACTTTAAACATTGATTTT
ATTTTTTATGTTTTTAGATGTTCATGTTTTTGAATGTTTTATTCGAATAATATAAAAAATA
AAATATATATTGACATGGATTAAACAAAGATATATATTATTCTATGTTGTATAAACAAA

TTGGCAAAATAGAGATGGAAGATAAAAATATGGTCAAAGTAATAAGAGTCTATGGTGA
ATGCCTA

>AX55 ***B. garinii***
GGGAGAGTAAGTTATTGCCAGGGTTTTTCTTTTATACTTTAAACATTGATTTTATTTTTTA
TGTTTTTAGATGTTTATATTATTTGAATGTTTTATTCAAATAATATAAAAAATAAAATATA
TATATTGACATGGATTAAACAAAGATATATATTATTCTATGTTGTATAAACAAATTGGC
AAAATAGAGATGGAAGATAAAAATATGGTCAAAGTAATAAGAGTCTATGGTGAATGCC

>AX56 ***B. afzelii***
GAGTTCGCGGGAGAGTAAGTTATTGCCAGGGTTTTTATTTTATACTTAAATCTTGAATTT
ATTTTTTAAATGTTTATATTATTTGAATGTTTTATTCAAATAATATAAAAAATAATATATA
TATTGACATGGATTAAACAAAGATATATATTATTCTATGTTGTATAAACAAATTGGCAA
AATAGAGATGGAAGATAAAAATATGGTCAAAGTAATAAGAGTCTATGGTGAATGCCTA

>AX65 ***B. garinii***
GAGTTCGCGGGAGAGTAAGTTATTGCCAGGGTTTTTCTTTTATACTTTAAACATTGATTTT
ATTTTTTATGTTTTTAGATGTTTATATTATTTGAATGTTTTATTCAAATAATATAAAAAAT
AAAATATATATATTGACATGGATTAAACAAAGATATATATTATTCTATGTTGTATAAAC
AAATTGGCAAAATAGAGATGGAAGATAAAAATATGGTCAAAGTAATAAGAGTCTATG
GTGAATGCC

>AX66 ***B. afzelii***
GAGTTCGCGGGAGAGTAAGTTATTGCCAGGGTTTTTATTTTATACTTTAAATCTTGAATTT
ATTTTTTAAATGTTTATATTATTTGAATAAAACATTCAAATAATATAAAAAATAATATAT
ATATTGACATGGATTAAACAAAGATATATATTATTCTATGTTGTATAAACAAATTGGCA
AAATAGAGATGGAAGATAAAAATATGGTCAAAGTAATAAGAGTCTATGGTGAATGCC

>AX69 ***B. afzelii***
TTGGAGTTCGCGGGAGAGTAAGTTATTGCCAGGGTTTTTATTTTATACTTTAAACCTTGA
ATTTATTTTTTAAATGTTTATATTATTTGAATAAAACATTCAAATAATATAAAAAATAAT
ATATATATTGACATGGATTAAACAAAGATATATATTATTTTATGTTGTATAAACAAATTG
GCAAAATAGAGATGGAAGATAAAAATATGGTCAAAGTAATAAGAGTCTATGGTGAATG
CCTAAAAAC

>AX72-2-2 ***B. valaisiana***
GTTCTGCGGGGAGAGTAAGTTATTGCCAGGGTTTTTATTTTGTACTTTAAACCTTAAATTT
ATTTTTTATATTTTTTTAATGTTCATGTTTTTGAATGTTTTATTCAAATAATGTAAAAAATA
AAATAGATATTGACATGGATTGAACAAAAGATATATATTATTTTATGTTGCATAAACAA
ATTGGCAAAATAGAGATGGAAGATAAAAATATGGTCAAAGTAATAAGAGTCTATGGTG
AATGCC

>AX72-2-8 ***B. afzelii***
GTTCGCGGGAGAGTAAGTTATTGCCAGGGTTTTTATTTTATACTTTAAACCTTGAATTTAT

TTTTTAAATGTTTATATTATTTGAATGGTTTTATATTCAAATAATATAAAAAATAATATAT
ATATTGACATGGATTAAACAAAGATATATATTATTCTATGTTGTATAAACAAATTGGCA
AAATAGAGATGGAAGATAAAAATATGGTCAAAGTAATAAGAGTCTATGGTTGAATGCC
T

>AX77-2-9 ***B. garinii***
GTTCGCGGGAGAGTAAGTTATTGCCAGGGTTTTTATTTTATACTTTAAACATTGATTTTAT
TTTTTATGTTTTTAGATGTTCATGTTTTTGAATGTTTTATTCGAATAATATAAAAAATAAA
ATATATATTGACATGGATTAAACAAAGATATATATTATTCTATGTTGTATAAACAAATTG
GCAAAATAGAGATGGAAGATAAAAATATGGTCAAAGTAATAAGAGTCTATGGTGAATG
CCTAAAAA

>AX77-2-14 ***B. afzelii***
TAGTTCGCGGGAGAGTAAGTTATTGCCAGGGTTTTTATTTTATACTTTAAATCTTGAATTT
ATTTTTTAAATGTTTATATTATTTGAATAAAACATTCAAATAATATAAAAAATAATATAT
ATATTGACATGGATTAAACAAAGATATATATTATTCTATGTTGTATAAACAAATTGGCA
AAATAGAGATGGAAGATAAAAATATGGTCAAAGTAATAAGAGTCTATGGTGAATGCCT
AAAAAGGGTGCTGGTAGTGGGGAGCTCTAACGTAGCACGGGTTAAGAGGGGTGTACTG
AACAGGGTGAAGGGAGACCAGAGGGTAACAGTAGAGGTCCAGTCAGGGAAGTGTATG
GTGAATGCCTACAG

>AX80-2-6 ***B. afzelii***
TCGCGGGAGAGTAAGTTATTGCCAGGGTTTTTATTTTATACTTTAAATCTTGAATTTATTT
TTTAAATGTTTATATTATTTGAATAAAACATTCAAATAATATAAAAAATAATATATATAT
TGACATGGATTAAACAAAGATATATTTATTCTA

>AX81-2-2 ***B. afzelii***
GAGTTCGCGGGAGAGTAAGTTATTGCCAGGGTTTTTATTTTATACTTTAAATCTTGAATTT
ATTTTTTAAATGTTTATATTATTTGAATGTTTTATTCAAATAATATAAAAAATAATATATA
TATTGACATGGATTAAACAAAGATATATATTATTCTATGTTGTATAAACAAATTGGCAA
AATAGAGATGGAAGATAAAAATATGGTCAAAGTAATAAGAGTCTATGGTGAATGCCTA
AAA
