# Supplementary material for: The AxBioTick Study: Borrelia Species and Tick-Borne Encephalitis Virus in Ticks, and Clinical Responses in Tick-Bitten Individuals on the Aland Islands, Finland
Source: Microorganisms. 2023 Apr 22;11(5):1100. doi: 10.3390/microorganisms11051100 (PMC10223457; doi:10.3390/microorganisms11051100)
Supplement: Supplementary file 1 [file microorganisms-11-01100-s001.zip › Supplementary Table S1.pdf]

Supplementary Table S1. *Borrelia* and Tick-borne encephalitis virus (TBEV) infected participants, eight-weeks (+/- 2 weeks) after tick-bite.

**Participants**								**Ticks**									
								**Inclusion tick**				**Additional ticks**				**Tick bites reported**	
Sex	Age	Diagnosis	Reported symtoms 8 weeks	Reported symtoms 4 months	S.con *Borrelia* C6 IgG1	S.con TBEV IgG	S.con TBV IgM	Stage	*Borrelia* PCR	TBEV PCR	*Borrelia* spp.	No. of collected additional ticks	*Borrelia* PCR	*Borrelia* spp.	TBEV PCR	Before	During
M	80	-	-	-	+	-	-	N	-	-		29	1/29	B.g	-	+	+
M	50	-	-	-	+	-	-	A	-	-		9	1/9	UT	-	+	+
F	61	-	-	-	+	-	-	N	-	-		3	-		-	+	+
M	73	-	a	-	+	+	-	N	-	-		2	-		-	+	+
M	69	-	-	-	+	-	-	N	+	-	B. a	-				+	+
M	44	-	-	-	+	-	-	L	-	-		1	-		-	+	+
F	67	-	-	-	+	-	-	N	+	-	B. a	8	2/8	UT/UT	-	+	+
M	72	-	-	-	+	-	-	A	+	-	B. v	-	-			+	-
F	63	-	-	-	+	-	-	N	-	-		1	1/1	UT	-	+	+
F	66	-	-	-	+	-	-	N	+	-	B. g	-				+	-
M	61	-	-	a	+	-	-	N	-	-		-				-	-
F	62	EM	EM	a,e	+	-	-	N	+	-	B. g	3	1/3	UT	-	-	+
F	63	EM	-	EM	+	-	-	N	-	-		2	2/2	UT/UT	-	+	+
F	60	-	b,c,d	N/A	+	-	-	A	-	-		2	2/2	UT/UT	-	+	+
M	72	EM	EM,g	-	-	-	-	N	-	-		21	7/21	B.a/B.a/B.v/ UT/UT/UT/ UT	-	+	+
F	65	EM	EM	a	-	-	-	N	+	-	B. a	-				+	+
F	68	EM	EM	-	-	-	-	N	-	-		-				+	+
M	50	-	f	N/A	-	+	+	N	-	-		-				+	-
F	74	-	-	-	-	+	-	A	+	-	UT	-				+	-
M	62	-	f,g,h,i,j,k,l	g,l	-	-	+	N	-	-		1	1/1	UT	-	+	+

Abbreviations: EM; *erythema migrans*, S.con; seroconversions, N/A; not available, B. a.; *Borrelia afzelii*; B. g.: *Borrelia garinii*; B. v.: *Borrelia valaisiana*, UT; untypeble a) myalgia/arthralgia, b) neck pain, c) dizziness, d) numbness, e) nonspecific, f) strong bite reaction, g) fatigue, h) chills, i) fever j) nausea, k) loss of appetite, l) headache Blank space; not analyzed
